# The Socio-Ecological Context of the Nutrition Transition in Indonesia: A Qualitative Investigation of Perspectives from Multi-Disciplinary Stakeholders

**DOI:** 10.3390/nu15010025

**Published:** 2022-12-21

**Authors:** Oyedolapo A. Anyanwu, Elena N. Naumova, Virginia R. Chomitz, Fang-Fang Zhang, Kenneth Chui, Martha I. Kartasurya, Sara C. Folta

**Affiliations:** 1 Friedman School of Nutrition Science and Policy, Tufts University, 150 Harrison Ave, Boston, MA 02111, USA; 2 Public Health & Community Medicine, School of Medicine, Tufts University, 136 Harrison Ave, Boston, MA 02111, USA; 3 Department of Public Health Nutrition, Universitas Diponegoro, Semarang 50275, Jawa Tengah, Indonesia

**Keywords:** nutrition transition, multi-disciplinary experts, dietary behavior, CVD risk factors, food environment, marine pollution

## Abstract

Background: Indonesia is undergoing a rapid nutrition transition—a shift in food consumption related to globalization, modernization, urbanization, and economic development—with potentially adverse impacts on diets, health, and the environment. This study sought to understand the perspectives of a multi-disciplinary group of experts on the effects of the nutrition transition on dietary behaviors, cardiovascular disease (CVD) risk factors, and the food environment in Indonesia. Methods: In-depth interviews were conducted online with 27 Indonesian experts, who are either healthcare providers, nutrition researchers, or environmental researchers. Interview question guides were developed based on a socio-ecological framework. We analyzed the data using deductive and inductive approaches. Results: Experts described a disconnect between awareness about and adherence to healthy diets among Indonesians. They highlighted a marked generational divide in food preferences between the younger population (<40y) and older population (40y+), due to the nutrition transition. Experts perceived that the nutrition transition has also resulted in more eating out, which promotes obesity, through the unhealthy menu offerings from restaurants. Experts also implied that traditional diets are no better than modern diets, due to unhealthy cooking practices, especially frying; suggesting that the combination of higher consumption of fast foods and unhealthy cooking practices may have worsened CVD risk factors in the population. Conclusion: Multi-disciplinary experts indicated that the nutrition transition has negatively impacted diets, health, and food environment in Indonesia. Our findings offer potential hypotheses that can be tested using quantitative approaches, to inform policy and the design of programs to reduce the adverse impacts of the nutrition transition in Indonesia.

## 1. Introduction

Nutrition transition, a process marked by changes in the dietary patterns of a population as a result of rapid economic development, urbanization and food systems transformation, is increasing the prevalence of chronic non-communicable diseases (NCDS) in lower-middle income countries (LMICs), including Indonesia [1,2,3,4]. Economic development means more employment opportunities, higher disposable income, better education, reduction in mortality due to infectious diseases and higher life-expectancy—a process better known in the literature as demographic and epidemiological transitions [2,5,6]. Economic development also means a shift from a mainly agrarian and labor-intensive economy to an industrial and service-oriented workforce, which promotes a sedentary lifestyle [7,8].

The nutrition transition presumably occurs in five stages: (1) hunter-gatherer and rummaging period; (2) famine period, marked by episodic food shortage; (3) receding famine stage, where societies reduce hunger through agricultural revolution; (4) emergence of diet-related chronic diseases, due to dietary patterns changing to a more westernized diets, with higher saturated fats from animal sources, refined carbohydrates and added sugar content and lower intake of whole grains, and (5) behavior change stage, marked by country-level approaches to reduce epidemic of chronic disease through consuming healthier diets [7,8,9]. It is hypothesized that in the future ideal society, lifestyle and dietary choices will tend towards stage 5 of the transition, with more active living, more consumption of fruits and vegetables and lower consumption of fats and highly processed foods [10,11]. While many high-income countries appear to be moving towards stage 5 of the nutrition transition, many LMICs are rapidly progressing to stage 4, which is characterized by higher prevalence of diet-related chronic diseases [11,12,13]. According to the Indonesian National Economic Survey (SUSENAS) reports, Indonesian diets have under gone rapid nutrition transitions, following the country’s accelerated economic growth in the 70s [14].

The global economic burden due to diet-related NCDs is expected to reach $47 trillion by 2030 [1,15] and currently, LMICs disproportionately bear 80% of all NCD-related deaths, which are preventable [1]. Specifically, Indonesia is experiencing a higher prevalence of cardiovascular disease (CVD) risk factors like hypertension and obesity [4,14]. CVD is the leading cause of mortality in Indonesia [16,17], and mortality due to CVD almost tripled from 9% in 1986 to 26% in 2001 [17]. In a national household survey to characterize the prevalence of CVD in Indonesia, Boedhi-Darmojo associated the rise in CVD risk factors to the westernization of diets, particularly in big cities. The globalization of food systems is an important trend contributing to nutrition transitions, resulting in the rise of supermarkets and fast food franchises, often in urban centers [1,18]. Although there is strong evidence linking processed foods with increased risk of developing chronic diseases [19,20,21], ‘imported’ foods, which are often highly processed, are preferred in many LMICs, in terms of convenience, since they are easier and faster to prepare, and many are ready-to-eat or ready-to-heat, compared to local, minimally processed foods [22]. In Indonesia, buying imported foods, or eating at fast food franchise like McDonald or KFC is associated with affluence and higher social class [23]. Fast-food franchises capitalize on increases in disposable income to aggressively market low nutrient but energy-dense foods in many urban locations [6,12]. However, relatively few studies from Indonesia have assessed awareness of and adherence to healthier diets, and most of these have used quantitative approaches [24,25].

Further, part of the health impact of the nutrition transition is the double burden of malnutrition (DBM) paradox, a condition in which undernutrition and overweight co-exist within one population. The rising prevalence in overweight in many LMICs is attributable to easier access to affordable ultra-processed foods and sugar-sweetened beverages in both urban centers and rural areas, in tandem with reduced physical activity at work and home and through greater reliance on labor-saving appliances [22]. Indonesia currently experiences high levels of the DBM [22]. Previous quantitative studies on the DBM in Indonesia have mainly focused on the demographic determinants [4,25,26,27]. There is a need for studies addressing socio-cultural contexts to inform the design of public health programs [25]. One multi-center qualitative study that utilized a socio-ecological framework to explore the impact of the nutrition transition on diets and physical activity across five countries, including Indonesia, reported greater reliance on western and convenience foods due to busier work schedules and indicated that socio-cultural determinants of obesity are generally understudied in the literature [23].

Additional studies are also needed to investigate the composition of traditional diets, cooking practices and their effects on CVD risk factors in Indonesia [14,28]. Our study team recently conducted a study to assess the cross-sectional relationship between dietary patterns and hypertension and obesity in a nationally representative sample of survey respondents in Indonesia. We found that both a ‘traditional’ dietary pattern marked by higher intake of fruits and vegetables and a ‘modern’ pattern characterized by more processed foods, significantly increased the odds for obesity [28]. Our findings suggest that how food is prepared is as important as the type of food being consumed. However, to our knowledge, this is the only study that investigated this. More studies are warranted to help confirm and contextualize these findings.

Bamberger et al. used a business-as-usual model to assess the impact of the nutrition transition on key environmental indicators in seven emerging countries, including Indonesia, from 2011 to 2030. They projected that the environmental impacts will be highest for Indonesia, with a clear trend for higher consumption of animal source foods (ASF), vegetable oils and fats for all the countries [29]. Authors also noted the dearth of literature assessing the effects of food system changes on the environment even though previous studies have warned of a potential increase in environmental impacts due to the nutrition transition [29].

Qualitative studies may help address many of these gaps by allowing us to understand some of the socio-cultural factors, including cultural beliefs towards food, cooking practices, and other contexts that impact dietary behaviors, health and the environment, in ways that quantitative approaches do not readily capture [30]. Our study contributes to the current literature by helping to bridge the knowledge gap about the socio-ecological context of the nutrition transition in Indonesia and how it impacts CVD risk factors, based on the multi-disciplinary perspectives of stakeholders. Our results also provide an evidence base to help inform policy guidelines and the design of health education programs tailored to different sub-populations.

## 2. Methods

### 2.1. Study Design and Conceptual Framework

**Figure 1:**
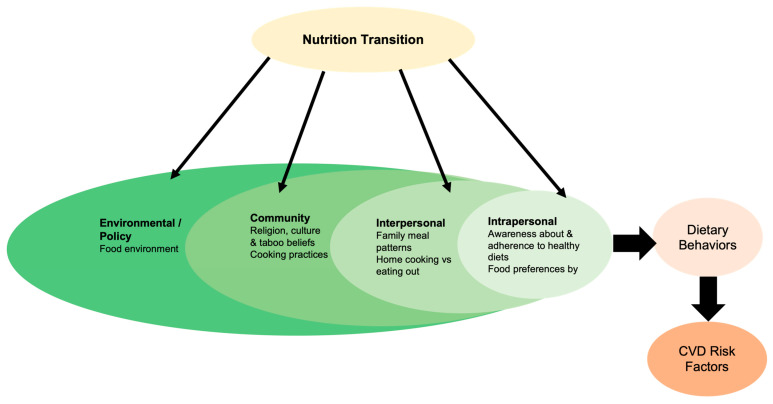
Socio-ecological model of the impacts of nutrition transition on dietary behaviors and CVD risk factors in Indonesia.

This study was as part of a larger theory-based, qualitative study to understand perspectives of a multi-disciplinary team of experts on diets, health and environment in Indonesia. In this paper, we addressed the impact of the nutrition transition on dietary behaviors and risk factors for CVD, such as hypertension and obesity, among the Indonesian population. We applied the Consolidated Criteria for Reporting Qualitative research (COREQ) guidelines in reporting this study [31] and used the socio-ecological model (SEM) as an organizing framework (Figure 1). The SEM allows for a more holistic and contextual examination of health behaviors by identifying multiple levels of influence (intrapersonal, interpersonal, community, and environment/global policies) on an individual’s health behavior [23]. Further, it helps us to identify which level or levels to address to bring about the desired change in behavior. The SEM is also amenable to integrating other theories and models. Hence, in recent years, there is a shift from single-level, individual-focused behavior change theories to broad, comprehensive frameworks to investigate health behaviors [32,33,34].

Based on the SEM, intrapersonal factors are those within an individual such as their knowledge, attitudes and beliefs that inform their health behaviors. At this level, interview questions addressed awareness about and adherence to healthy diets generally, and to the Indonesian dietary guidelines (IDGs) particularly. Questions also assessed the impact of the nutrition transition on individual food preferences. Interpersonal factors are external sources of influence on an individual’s health behaviors, such as from parents, peers or significant others. Here, questions addressed the typical family meal patterns and further examined the influence of the nutrition transition on social dynamics. The community- and regional-level factors are sociocultural factors, such as norms, beliefs and cultural practices commonly found across individuals and families. Within this level, we investigated the role of religion and cultural beliefs on food choice and sought to understand the impact of the nutrition transition on cultural meal patterns. The global and policy level factors are upstream factors that transcend community and regional boundaries. At this level, we investigated the changes in the food environment due to the nutrition transition.

### 2.2. Description of Research Team

The composition of the research team has been previously described [35]. The study was conducted by a multidisciplinary team of Ph.D. level academic researchers from Tufts University, Boston MA and Universitas Diponegoro (UNDIP), Semarang Indonesia, who have many years of teaching and research experience (ENN, VRC, FFZ, KC, MIK and SCF), and one doctoral student (OAA), who coordinated the study as part of her doctoral dissertation project. The research team combined expertise from the fields of nutrition, epidemiology, public health and environmental health. The senior author of the study (SCF) is a qualitative expert with extensive publications using the qualitative approach. All but one of the research team members are of the female gender. Two study team members are from LMICs: OAA is Nigerian, and MIK is Indonesian. MIK ensured the cultural relevance of the questions and helped with the interpretation of our findings.

### 2.3. Location, Recruitment and Interview Procedures

The recruitment, sampling strategies and interview procedures have been previously described [35]. The first author (OAA) and a trained research assistant conducted in-depth interviews online with a purposive sample of key informants from across Indonesia (*n* = 27), who are either healthcare providers, public health nutrition researchers or environmental researchers, between April and June 2021, a period when Indonesia had just emerged from the first wave of the COVID-19 pandemic and when the social restriction policies imposed by the Indonesian government were starting to ease. Proficiency in the English language was a criterion to participate in the study. This was due to a lot of restrictions placed on the type of research that the institutional review boards (IRBs) of universities would allow during the COVID-19 pandemic. The IRB protocol for ensuring the accuracy of translated materials would have meant a longer wait period than the funding cycle for the study allowed for. Further, coordinating the logistics for appropriate translation services was going to be complex and difficult because of lockdowns and restrictions on mobility in both the United States and Indonesia. Thus, we were strategic in the recruitment process to include experts who not only have depth of knowledge and experience in the topic areas, but also have fluency in English to communicate their perspectives clearly.

Invitations to participate in the study were sent by emails and those who respond to the emails were sent a link to fill out a demographic questionnaire. Healthcare providers were recruited based on their familiarity with the dietary needs and behaviors of the adult population in Indonesia. Public health nutrition researchers were recruited for their expertise in food environment and nutrition policy in Indonesia. Environmental researchers were selected for their expertise on the impacts of environmental factors on the quality and availability of heart-healthy foods such as fish, fruits, and vegetables. A lack of internet access was an exclusion criterion since the interviews were conducted online. A study team member read the informed consent document to each participant by WhatsApp audio call and received their verbal consent prior to each interview session. The sample size was adequate to capture a range of perspectives and reached saturation on the impacts of the nutrition transition on the topics addressed at the different levels of the framework. We determined that we have reached saturation when no new information or perspective was shared that had not been previously expressed [30,36]. Recruitment followed a snowball recruitment strategy. Two members of the study team (ENN and MIK) recommended the first set of informants that were recruited and interviewed. We then asked these informants to provide additional contacts at the end of their interview sessions The study was approved by the institutional review boards (IRB) of Tufts University and the Universitas Diponegoro (UNDIP) Indonesia.

A semi-structured interview guide was developed, using the SEM as a framework, by a multidisciplinary team consisting of experts in the field of qualitative methods and epidemiology, as well as two collaborators who are public health nutritionists from UNDIP in Indonesia. Study collaborators from UNDIP reviewed the question guide for cultural relevance. We then pilot-tested with two Indonesians in relevant areas of expertise, who share similar demographic characteristics with those we recruited for the study, to ensure that the questions on the guides were interpreted as intended. We iteratively refined the interview guide as the study progressed by eliminating and streamlining questions as needed, for better clarity. The final interview questions guide is provided as an Appendix A. Before the start of each interview session, the interviewer reminded the informant of the purpose of the study and their rights as an informant and explained that the research was part of a doctoral dissertation project. Each interview session lasted between 45 to 60 min and was recorded. In addition, the interviewer took notes of the non-verbal responses of the informant. Only the interviewer and informant were present during each session. At the end of each session, the interviewer provided a general summary to validate perceptions of the main points.

### 2.4. Data Analysis

Recorded data from the in-depth interviews were transcribed verbatim, using the services of an online transcription company (GoTranscript LTD. Middlesex, United Kingdom). Transcripts were crosschecked against the audio recordings and notes taken during the sessions. A codebook was developed iteratively from the transcripts, using a qualitative content analysis approach [30]. Analysis was primarily deductive based on the study framework, although some themes emerged inductively. To establish intercoder reliability, two study team members independently coded three randomly selected transcripts, one from each category of experts, using the codebook. No major issues were identified in code agreement except the need to better clarify the description of some codes. Once the minor discrepancies were addressed, the intercoder reliability score was >90% agreement and at least 0.75 on the kappa coefficient for all codes. With the codebook refined and performing well, we proceeded with the full coding of all the transcripts. The NVivo software (Version 12 QSR International; 2018) was used to create codes and assist with analysis. In the deductive analysis phase, we closely explored each code to understand the ideas being conveyed by running word searches and text queries. We conducted multiple rounds of coding to meaningfully categorize and combine ideas, thoughts and views. For the inductive analysis, we developed codes for salient ideas that emerged from the data that did not fit into the framework. We followed the same iterative process of exploration and categorization of codes as was done at the deductive phase. Categories that reflect similar ideas were combined as themes, and topics addressed under each theme were classified as sub-themes. Emerging themes were iteratively reviewed and refined and later verified by study collaborators in Indonesia [30].

## 3. Results

Table 1 shows the demographic characteristics of the experts interviewed. The majority were women (*n* = 19). Breakdown by ethnicity was predominantly Javanese (*n* = 14) and mean age was 46 years (SD 11.9). Among all experts, 13 hold a PhD degree, 6 have a professional medical or nutritional qualification, 5 hold a Master’s degree, and the remaining 3 have a Bachelor’s degree. When categorized by years of working experience, a greater proportion of experts who are public health nutrition and environmental researchers had at least 11 years working experience, while the majority of those who are healthcare providers had worked less than 5 years.

## 4. Themes

Experts’ perspectives evoked seven themes across the four levels of the socio-ecological framework. At the individual level, the two themes were related to awareness about and adherence to healthy diets, and the impact of the nutrition transition on dietary preferences and health outcomes among the population. At the interpersonal level, themes addressed the traditional meal patterns of families, and changes in family and social eating patterns due to nutrition transition. At the community/regional level, two themes highlighted the impacts of cultural factors and nutrition transition on food choice and cultural meal patterns. Finally, at the environmental / policy level, one theme addressed the impact of the nutrition transition on the food environment. Table 2 summarizes key findings.

### 4.1. Individual-Level Theme 1: Awareness about and Adherence to a Healthy Diet

Experts described the level of awareness about and adherence to healthy diets by the general population. Overall, experts noted that while many are aware of what constitutes a healthy diet, adherence is generally low. Vegetable consumption was most often referenced by experts in relation to people’s awareness about healthy diets, but consumption trends vary based on the ethnicity, age group, education level or socio-economic status (SES). The experts said that although people generally recognize the health benefits of vegetable consumption, they tend not to eat vegetables because they do not taste good. Experts further noted that people with low income and low education levels tend to think more about affordability and satiety of food than the nutritional aspects.

*“If I may add, I think nowadays, more people have gained awareness, but there’s this thing that they know it’s healthy to eat, but they do not eat it because they know vegetables are not delicious.”*

(22-year-old female, a healthcare provider).

Experts discussed awareness about and adherence to the Indonesian Dietary Guidelines (IDGs) and compared the old dietary guidelines (from 1929 to 1995) to the current IDGs (from 1995 to present). The old IDGs focused on the macronutrient composition of a meal, while the new guidelines focus mainly on nutritional balance and emphasize variety, portion size, food safety and physical activity. Experts implied that the messaging of the old IDGs was comparatively simpler to remember by the general population than the new IDGs, which were described as more complex and harder for the population to follow. In terms of awareness about and adherence to the IDGs, informants stressed the popularity of the slogan for the old guidelines “*Four essential but five to be perfect*,” while they noted that fewer, mainly educated folks, know the current ones. In addition to reporting low awareness of the current IDGs, participants also described low adherence to them among the general population.

*“When I was in elementary school, it’s very popular and they’re easy to digest, easy to understand, and then now is being revised into balanced nutrition. Maybe it’s less popular than Empat Sehat Lima Sempurna. I think many people in the same age as me or older, they still remember Empat Sehat Lima Sempurna.”*

(45-year-old male, an environmental researcher).

Experts identified several barriers to following the current IDGs, including: affordability, low awareness, low literacy, complexity of the new guidelines compared to old, tastes and preferences. When people do not have the means, they will only go for what they can afford. For people in the rural areas, it becomes too complex for them to remember what portion sizes are recommended for the different food groups. For many Indonesians, their food choices are driven by tastes and preferences for certain types of foods that do not necessarily conform with the IDGs.

### 4.2. Individual-Level Theme 2: Impact of the Nutrition Transition on Dietary Preferences and Health Outcomes

Experts described the nutrition transition as eating “junk food” or “fast food” from the West or other Asian countries like Korea, Japan and Thailand. They suggested that there is a marked generational divide in food taste and preferences and implied that the younger generation usually prefers the western-type foods, while the older generation will tend to prefer the more traditional dishes. Experts highlighted health consciousness as a major reason for the nutritional divide between age-groups. Their perspective was that the older that people get, the more health conscious they become, and older people may also have one or more chronic disease diagnosis, which may motivate them to make dietary choices that suit their health situation. For the younger generation, although some of them may be aware of the risks involved in consuming certain types of foods, they do not care so much, because they do not consider themselves to be susceptible to such health problems.

*“Yes, I think all of the people, depend on age, when the people, youth generation, it’s 17 till 30 years old, they ignore about the fat, and then the vegetable oils, et cetera, but when people in 40 above age, they’re more selective to eating the food because they have some disease, like diabetes mellitus, and then cardio, heart attack. They more think when to consume some food.”*

(40-year-old female, an environmental researcher).

Experts explained that the nutritional divide does not seem to apply to beverages however, since Indonesians whether young or old, still like the traditional beverages, although special mention was made about the proliferation of new brands of tea and coffee, that are different from the traditional ones:

*“I remember that previously we only drink coffee, milk, and tea but now there are many package drinks. For instance, now it’s very popular Thai tea drinks and bubble drinks. The tea are mixed with condensed milk sometimes. They have a small amount of fresh milk, but lots of condensed milks and sugar… Also, there is bubble that made from tapioca flour inside and also provide flavor like strawberry or coffee, or chocolate. This kind of new foods and drinks is very popular now.”*

(44-year-old female, a public health nutrition researcher).

Experts expressed concern at the rise in diet-related chronic diseases among the population due to the nutrition transition, while infectious diseases and undernutrition still constitute significant public health burdens. They identified children, pregnant women and the elderly as having higher risks for undernutrition, while obesity cuts across all age-groups and SES levels.

*“We have three burdens now. We still have infection, that high number of tuberculosis. We have a malnutrition is under and over nutrition. Then now, we increase number on non-communicable diseases. Therefore, heart disease, cardiovascular, hypertensive and diabetic. Wow make us!”*

(65-year-old female, a healthcare provider).

### 4.3. Family and Interpersonal Level Theme: Changes in Family and Social Eating Patterns Due to the Nutrition Transition

Experts described some changes in family meals patterns due to nutrition transition. For instance, eating out with family and friends is becoming an integral part of the Indonesian culture, especially in the urban centers. Information about new restaurants spread by family and friends’ connections during social gatherings or from one friend to another.

Experts indicated that there are plenty of restaurant options in Indonesia to support the emerging culture of eating out: street foods by food hawkers, local food shops, fast food chains and high-end restaurants. Experts also described the development of family-owned microenterprises, whereby some traditional families that cook regularly, offer catering services to families or individuals who do not have the time or do not know how to cook. With these many options, eating out is often affordable. Further, experts noted that people choose to eat out for networking and conducting business meetings. Eating out is also considered a time of relaxing and rest for families. For the younger population, it affords an opportunity to get away from the older adults and hang out with peers. One expert put all these together very succinctly:

*“… We solve a lot of business problem by meeting outside, by having lunch or dinner, especially, if you really want to have a special deal… In fact, there was a time when I never cook because the reason is why because sometimes it’s cheaper to buy food rather than you cook in Indonesia.”*

(59-year-old male, an environmental researcher).

Additionally, experts noted the influence of the nutrition transition on children feeding practices. Before, parents had more control over what the children ate but in recent times, if the children do not want to eat certain foods, parents easily comply, and often stock unhealthy options at home.

*“Maybe it’s also a part of nutrition transition because the way people, parents are parenting their children, I think now it’s also changed. compared to my era, my mother always provide milk and then vegetable and I have to finish otherwise, my mom will be angry [laughs] but now I think in the younger parents they just follow what the children want. If the children don’t want to eat fish or vegetables and they just choose instant noodle, for example, ‘Okay, you can have it,’ and it’s always available at home.”*

(44-year-old female, a public health nutrition researcher).

Experts highlighted some specific ways that eating out impacts dietary behaviors and ultimately health outcomes. For instance, eating out changes people’s food choice and preferences over time, due to the availability of more options and encourages overeating through bigger portion sizes that are cheap.

*“… Teenagers, especially—under 20, they prefer to eat with their friends… they told me that if they have to eat together, they prefer to choose the big size so they can eat more and why they choose that big size because sometimes the price is not as expensive as if they choose the regular one.”*

(38-year-old female, a healthcare provider).

Experts also noted that snacks are abundantly available in most social contexts and implied that this may be promoting more snacking behavior, which contributes to obesity:

*“If you work in the office, you will eat—if you have a meeting, you will have a snack box, always like that. In the content of the snacks box is one sweet stuff, like cookies, not cookies actually, cake, quite heavy cake, salty one. [chuckles] Then probably crackers and sometime juice. Very heavy, actually, very heavy snack box we have. Imagine, if you have meeting every single day, every day you will have this kind”*

(59-year-old male, an environmental researcher).

### 4.4. Sub-Theme: Home-Cooked Meals Versus Meals Prepared Outside the Home

Experts described low level of home-cooking compared to previous generations. Time constraint was a major factor identified, particularly for urban dwellers. They also suggested that the presence of more women in the work force contributes to this growing disinclination to cook meals at home.

*“Nowadays, many people, especially in Indonesia, mothers because there is a still gender situation as well about cooking, have less time than before. Less time for buying, less time for selecting foods, less time for cook. I think it is one issue, it’s related to convenience and especially time-saving foods.”*

(52-year-old male, a public health nutrition researcher).

Experts further clarified that whether a family consumes home-cooked meals would also depend on economic status. Families who can afford it hire domestic helps (*house assistants*) to prepare the food at home when the mother works. Otherwise, they will order food from outside, while the low-income families will cook at home.

*“In Indonesia, actually, even the mother that works in the office, they will have their assistant at home that sometimes live together or just come in the morning and then go back to their home in the evening. That assistant will cook the home food every day.”*

(38-year-old female, a healthcare provider).

Other factors related to less home-cooking include the elaborate nature of many Indonesian dishes, which are complex and time consuming to prepare, and lack of ready availability of some ingredients. Additionally, many people in Indonesia do not know how to cook some of these complex dishes and some of the cooking utensils are expensive.

*“Also, maybe the cooking skills because sometimes I also ask, “Why you didn’t prepare food for your kids?” “I don’t know how to cook nice food for children,” something like that. For me also, I don’t have complete tools for baking. So, if I want to eat cake I always order from outside.”*

(44-year-old female, a public health nutrition researcher).

### 4.5. Community and Regional Level Theme 1: Cultural Meal Patterns and Their Health Effects

Experts described some cultural and religious practices, including taboo beliefs, that are important drivers of dietary behaviors in Indonesia. For instance, consumption of pork and alcohol is a taboo for all the Muslim ethnic groups; the Hindu population will not consume red meat, but chicken and fish are allowed. Moreover, experts noted that taboo beliefs may lead to the restriction of some healthy foods with important nutrition content for some vulnerable populations like children, pregnant women and teenage girls.

*“Taboo food is a food that are not consumed because of certain belief in this food. In my opinion that is a dietary restriction. For example, the breastfeeding mom or pregnancy mom, they avoid to eat seafood and any animal protein sources because their belief that for a breastfeeding mom, if they eat seafood, it will make the milk stingy, something like that… You know, we have to straighten it out considering that sometimes, seafood have a high nutritional content.”*

(54-year-old female, a public health nutrition researcher).

Experts suggested that there is no difference between traditional foods and western foods in terms of healthfulness because of the way many traditional foods are prepared. They identified frying, repeated use of the same oil to fry foods, and the use of coconut milk as having the most significant influence on CVD risk factors in Indonesia. Experts explained that even foods considered healthy, like vegetables, are consumed in low amounts and often coated with wheat batter and fried, which reduces the healthfulness.

*“I’m worried about the fried because in Indonesia, maybe in every day, they will fry their food… That’s why in Indonesia, maybe the prevalence of cardiovascular diseases increased every year because a lot of people eat fried food.”*

(40-year-old female, a public health nutrition researcher).

### 4.6. Community and Regional Level Theme 2: Influence of the Nutrition Transition on Cultural Meal Patterns

Experts highlighted some evolution in traditional staples due to nutrition transition that is affecting food tastes and preferences, including in the rural areas, where people would typically cook traditional meals. Experts expressed concern about ultra-processed foods and the increasing use of some condiments, preservatives, food additives and taste enhancers by the food industry, which encourage higher caloric consumption, and promote obesity.

*“I think the high ultra-processed food been used in their dietary, in their food, like instant noodle…canned sardines, something like that, but also, in Indonesia, there are some instant spices that before, we use-- garlic, we use anything that’s from natural ingredient. Right now, industry provide the instant spices and have natrium added in it.”*

(54-year-old female, a public health nutrition researcher).

However, some experts perceived the nutrition transition positively. It is considered “innovative” and beneficial in terms of access to more food choices that are particularly suitable for working individuals in terms of convenience.

*“I mean, with the information’s related on how convenient this food is, on how nutritious and how good the taste is, and also it can fulfill the need to try for something new. To some extent, I don’t think the transition is necessarily negative… for me, it is a good thing. There are some innovation because of this. For example, there is a tempeh burger. You have a burger, but it is a tempeh inside.”*

(52-year-old male, a public health nutrition researcher).

### 4.7. Environmental Level Theme: Impact of the Nutrition Transition on the Food Environment

Experts noted the proliferation of foreign food franchises and described the marketing strategies of the food industry to increase patronage. For example, experts explained that fast food chains customize their menu offerings to mimic traditional meal patterns to better suit consumer tastes and preferences. Additionally, there is a rise in the number of online food platforms in recent times, due to technological advancement and growth in internet use, which make fast foods more accessible and affordable for every social class. Experts also noted that regional differences in food availability is gradually being eroded since traditional Indonesian dishes from different regions can be ordered from anywhere.

*“Yes, available to everyone. Everyone can make it. Not only expensive restaurant. Everyone can make it and then with the application we just look for it in the application so we can buy it… Everybody or everyone in Indonesia can reach, know about a burger, not only for the rich, for the expensive one. The cheap one, we have burger too.”*

(36-year-old female, a healthcare provider).

Experts described the nutrient composition of fast foods and restaurant menus in Indonesia as largely unhealthy because they are packed with refined carbohydrates, fat, salt, sugar and high cholesterol and are frequently accompanied by sugar-sweetened beverages and low intake of fruits and vegetables. Experts expressed concern that since people’s food choice is often dictated by what the restaurant offers, unhealthy options gradually become the norm, as people get used to eating the foods available at the restaurants.

*“When we choose the restaurant, we cannot choose any kind of food, we’ll just choose what they serve. Usually, they serve, honestly, unhealthy food. It’s all fried, it’s all processed meat, and then it’s all instant and a lot of oils, a lot of grease, a lot of fat. If we want to have healthier food, it tends to be more expensive. I think only a little part of people can access that food.”*

(37-year-old female, a public health nutrition researcher).

Yet, people might still prefer fast food chains (like KFC, McDonalds) to local street restaurants (Kaki Lima), because fast food chains have more hygiene protocols in place, even though their menu options offer less variety. However, experts felt that raising the standard of hygiene for local street restaurants would also have impact on the price of foods and become non-affordable, for low-income population.

*“Kaki lima is like a restaurant with a tent in the side of street, do you know? We cannot sure about where they got the water. Fast food is better than kaki lima. Fast food more hygiene and good quality. I think the level of diversity is less because we can buy only chicken, rice, burger, French fries. Approximately, only that choices in fast food. In kaki lima, we have so many choices. From vegetables, there is some kaki lima have 50 menus.”*

(36-year-old female, a healthcare provider).

### 4.8. Sub-Theme: Influence of Television Advertising and Social Media

Experts illustrated how restaurants and the food industry use television advertising and social media messaging to influence food trends, tastes and preferences, often toward higher purchase or consumption of unhealthy food options. For example, these media channels provide information on promotions and discounts, and feature demonstration videos on new food trends, menus, recipes and so on. When people, especially the younger generation, learn about these new foods, they want to try them out either by buying in the restaurant or by preparing it at home.

*“Yes, I think mothers easily get information. They can browse the internet. Then advertisement in television and in social media. The mother can practice her information, and can maybe impress… now, mother has changed from how to cook something good for her family… So, I think it’s better in past.”*

(36-year-old female, a healthcare provider).

Experts also described the additional role of food bloggers as a big influence on food tastes and preferences of Indonesians:

*“It can confuse people. I think you understand this, now it is the era of the death of expertise. You learn nutrition for how many years, I don’t know, 12 years maybe, but your comments will be easily wiped out by comments which does not really have an education basis at all.”*

(52-year-old male, a public health nutrition researcher).

## 5. Discussion

This study sought to broaden the currently sparse literature on the impacts of the nutrition transition on dietary behaviors and CVD risk factors among Indonesians, based on a socio-ecological framework. We conducted remote in-depth interviews with a multi-disciplinary group of Indonesian experts in the fields of healthcare, public health nutrition and the environment and analyzed themes using a qualitative content analysis approach.

At the individual level, experts’ perspectives revealed a discrepancy between awareness about and adherence to healthy diets, as well as low awareness and adherence to the current IDGs. This view is consistent with the few previous quantitative studies from Indonesia on awareness about and adherence to healthy diets [24,25]. Usfar and Fahmida reported low adherence to the 1995 IDGs messaging to consume some key nutrients and recommended age-specific guidelines and improved messaging to address higher in-take of fiber-rich foods like fruits and vegetables [24]. Experts also indicated a generational divide by age-group for food preferences, due to the nutrition transition and suggested that the health consciousness of the older population may be the main driver for this. This view contrasts with the findings of Lipoeto et al. [14] who found similar tastes in food preferences among the older and younger Indonesian participants interviewed in their study. The difference could be due to lapse in time since 2008, when their study was conducted. Additionally, their study was conducted in a more suburban and rural location, and captured only one region (West Sumatra), while our study experts come from diverse regions and ethnicities. Moreover, our results concur with the findings of other recent quantitative studies on socio-demographic determinants of dietary patterns in Indonesia that reveal a similar pattern of generational divide in food preferences [28,37]. In addition, experts implied that the nutrition transition is exacerbating the problem of multiple burdens of disease in Indonesia. This finding adds depth and richness to previous quantitative studies on the double-burden paradox in Indonesia [25,38], by specifying that people with low SES, and low education levels are more concerned with having enough food than about the nutritional content of food. Thus, they are usually striving for satiety, often through consumption of low-nutrient but high-calorie foods, which may lead to higher rates of undernutrition and overweight in vulnerable sub-populations.

At the interpersonal level, experts indicated that there is a growing trend to eat out and described many barriers to home-cooking. Experts evoked one important factor related to this—the presence of more and more women in the work force. In homes where the mother works, she may not be able to cook for the family, which could imply more intake of fast or convenience foods than home-cooked meals. Another barrier to home cooking they highlighted was lack of cooking skills and lack of cooking equipment. This is in line with the results of a qualitative study in which the authors found low willingness to cook among low-income participants who lacked kitchen equipment [39]. The literature has been consistent on the healthfulness of home-cooked meals compared to away from home meals. While there are important economic and social gains to more women in the workforce, the current trend of low home-cooking and higher intake of away from home foods is likely to grow, with lasting adverse impacts on the population health [40,41,42]. Further, experts revealed that nutrition transition contributes to obesity in the population by increasing snacking behaviors, with the constant availability of snacks in most social contexts, including home, schools, and workplaces. This view is consistent with the findings of a recent quantitative study on the relationship between habitual snack consumption and the prevalence of overweight among adolescents in the Tasikmalaya region of Indonesia [43]. Similarly, Andarwulan and colleagues used 2-day weighed food record to estimate food consumption pattern and intake of sugar, salt and fat in South Jakarta, and identified beverages and snacks consumption as the main sources of added sugar among respondents [44].

At the community level, experts suggested that the Indonesian food culture is rooted in religion and taboo beliefs and implied that these cultural beliefs are pervasive among the Indonesian society. According to our experts, these factors not only influence food choices, but they contribute to dietary restrictions and ultimately, may impact the nutritional outcomes of vulnerable sub-populations like children, teenagers and pregnant women. This view is consistent with previous literature on the role of religion and cultural factors on dietary behaviors in Indonesia [4,24]. Our findings extend the current literature by illustrating the context for some of the cultural beliefs and behaviors regarding food. For instance, experts explained that the physical environment, such as whether people are living in mountainous regions which are remote, may influence the kind of animals available as protein sources. While experts noted a gradual shift back to consuming traditional foods, the consensus was that there is no difference in the healthfulness of traditional foods compared to modern foods in terms of their nutritional contents. This is in line with the findings of Anyanwu et al., earlier cited, in which we compared a modern dietary pattern with a traditional dietary pattern among Indonesian adults [28]. Frying foods, including vegetables; high use of coconut milk and oil; and the use of the same oil several times, are some of the cooking practices identified by experts as likely driving the high prevalence of CVD risk factors in the Indonesian population. This perspective is supported by the few available literature on the nutritional composition of traditional meals in Indonesia [13,45]. In a quantitative study assessing the dietary quality of traditional diets among women in two regions of Indonesia using 2-day repeated 24 h food recall, authors reported a low Healthy Eating Index (HEI) score of <51 by 99% of study participants [45].

At the environmental and policy level, experts highlighted the proliferation of foreign food franchises that tend to mimic Indonesian traditional meal patterns, alongside a rise in the number of online food platforms, and implied that fast food is becoming more accessible and affordable to every social class, unlike in previous decades. Moreover, experts expressed concern that the mostly unhealthy menu offerings of restaurants and fast-food places in Indonesia may have increased CVD risk factors in the population, particularly since healthier menu offerings are often not affordable. Previous literature on the impacts of restaurant and fast-food meals on health outcomes in Indonesia corroborates this [1,4,12,46]. Andawulan and colleagues reported that meals from restaurants, street and fast-food are the major food sources contributing to salt and fat intake in their Indonesian study sample [44]. Additionally, in a recent randomized controlled-feeding trial to assess the quality and cost of a low sodium and potassium-rich diet compared to a typical Indonesian diet, authors found that while the potassium-rich diet is closely related to higher diet quality, the cost is higher, which may inhibit intake [46]. Our study adds context to and extends these findings by articulating some perceived benefits of nutrition transition among Indonesians. One is the convenience of ready-to eat and ready-to-heat foods for working parents and another is the general perception of better food safety of meals from western fast-food franchises compared to local street restaurants. These perceptions of benefits suggest that adherence to unhealthy foods from the food industry will likely continue unabated and intensify the negative health impacts of the nutrition transition in Indonesia.

This study has some limitations, which we addressed as much as possible to improve the validity of our approach. We interviewed researchers and health professionals, a greater proportion of whom are doctoral degree holders, with expertise in relevant fields, to provide their perspectives on how the nutrition transition has affected dietary behaviors, CVD risk factors and the food environment. We acknowledge that each expert would have their own biases and that their perspectives may not accurately represent the perspectives or realities of the general Indonesian population. However, purposively recruiting a variety of experts, to obtain a blend of perspectives on the different topics addressed, was also part of our study process to attain validity. Additionally, we recruited an adequate sample size for each category of experts to ensure that we achieve thematic saturation, which we did. We also shared our findings with a study collaborator from Indonesia to corroborate our findings. Nevertheless, due to our purposive approach to gain in-depth perspectives from multidisciplinary experts, this study was not able to address the perspectives from different socioeconomic levels. Future qualitative studies using focus groups and ethnographic approaches may consider recruiting a more diverse audience to better understand the lived experiences of the general population. Additionally, fluency in English was an inclusion criterion to participate in the study. While many of our participants are highly educated and have enough fluency in English to participate in the study, we anticipated that an impromptu expressing of their thoughts and ideas in a second language might still be a challenge. To address this and ensure that our participants present their perspectives as clearly and as detailed as possible, we sent the interview questions to them ahead of time, so they could provide clear and well reflected perspectives. Both their written and verbal responses were analyzed as part of our findings. Finally, the interviews were conducted remotely, online. Although the interviews were conducted with video on, and interviewers took note of the non-verbal cues from participants as much as possible, we acknowledge that we may have missed out on other non-verbal cues of in-person meetings, that could have provided a richer meaning and context to our findings than captured in the more static online environment. The socio-ecological framework employed to develop the interview question guide and analyze the themes allowed us to cover a broad scope of topics relating to the impacts of the nutrition transition along each level of the framework. Additionally, by recruiting experts from different fields, we gained in-depth, multi-disciplinary perspectives on the effects of the nutrition transition on each of the topics addressed. Moreso, the framework used will facilitate better targeting of interventions and programs to meet the needs of specific vulnerable sub-population at each level.

### Implications for Policy and Directions for Future Study

Based on the empirical evidence drawn from the multi-disciplinary perspectives of the experts interviewed on the impacts of nutrition transition in Indonesia, we offer some tentative policy recommendations and highlight directions for future research.

Our findings suggest a disconnect between awareness about and adherence to healthy diets. This disconnect may be linked to the fact that, as experts explained, how Indonesians define a healthy diet goes beyond focusing on nutrients, and include other components of people’s lived experiences, including their religious and social beliefs. Thus, future revisions of the Indonesian dietary guidelines might incorporate messaging that addresses socio-cultural aspects of nutrition in addition to nutritional contents. Dietary guidelines may also incorporate messaging targeted at the younger age-group, that are vulnerable to higher consumption of unhealthy diets, to mitigate the early onset of diet-related chronic illnesses. More empirical studies using both qualitative and quantitative approaches are warranted to investigate the impacts of the nutrition transition on the CVD risk factors in Indonesia. Such studies could potentially address differential effects by age-group.

Our results also indicated that mothers’ influence on family food preferences might be waning due to: lower inclination to cook meals at home; children’s food preferences influencing more what meals are taken at home and the growing dependence on domestic helps by some families. Yet, experts noted that mothers are usually the focus of nutrition interventions programs. We recommend that rather than just focusing on mothers as the priority audience of nutrition interventions, it might be worthwhile to use a whole family approach. Such interventions may also target domestic helpers, as they play a key role in shaping dietary intake of not just the children but the whole family. More studies assessing the long-term impacts of nutrition transition on family eating patterns are needed to help inform the design of nutrition interventions for families in Indonesia. Such study could incorporate mixed methods approaches to address the changing dynamics of infant and children feeding practices and the role of domestic helps in influencing household dietary patterns.

Further, our findings implied that taboo beliefs are deeply entrenched in the culture, fostering greater risks for nutritional deficiencies among vulnerable populations. Thus, nutrition interventions for Indonesia should address these beliefs in a culturally appropriate way, in order to promote behavior change. Given the dearth of literature on this topic, we recommend more studies exploring the direct effects of religion and taboo beliefs on nutritional outcomes for specific sub-populations in Indonesia. Our results also suggested that due to sub-optimal cooking practices, traditional meals are comparable to non-traditional meals in terms of their unhealthfulness. Additional studies are needed to investigate the health impacts of the changing composition of traditional diets in Indonesia, to inform policy guidelines and the design of nutrition programs to improve cooking practices.

Additionally, the findings of this study suggest that the nutrition transition is rapidly changing the food environment in Indonesia. Experts suggested that the local street restaurants (Kaki-limas) have more food options compared to western fast-food franchises, but that food safety is a concern. This may be an important sector for health programs to focus on, to increase hygiene and food safety awareness. Nutrition education programs may also target this sector, to increase the healthfulness of their menu offerings.

## 6. Conclusions

This study synthesized the perspectives of a multi-disciplinary group of researchers and health professionals to develop themes on the multi-faceted impacts of nutrition transition on dietary behaviors, CVD risk factors and the food environment in Indonesia. Our findings suggest that the nutrition transition may have worsened health conditions, particularly increasing risk factors for CVD in the Indonesian population. Experts’ views revealed that the messaging of the current Indonesian dietary guidelines could be made more accessible, particularly to sub-populations with low education level. Further, our results suggest that Indonesians could benefit from nutrition education programs to promote home-cooking and improve cooking practices. However, more national-level quantitative studies using gold standard dietary assessment methods are needed to corroborate our findings and better clarify the magnitude of and mechanisms by which dietary changes due to nutrition transition influence CVD risk factors, and the food environment in Indonesia.

## Figures and Tables

**Table 1:** Demographic Characteristics of Expert Informants.

Characteristics	Total
**Expert Informants**	**27**
Nutrition/Public Health Researchers	**10**
Healthcare Providers	**8**
Environmental Researchers	**9**
**Age (Yr) mean, (SD)**	**46.1 (11.9)**
**Gender, *n***	
Female	**19**
Male	**8**
**Ethnicity, *n***	
Javanese	**14**
Sundanese	**2**
Batak	**2**
Buginese	**2**
Sulawesi	**2**
Lampungenese	**1**
Chinese-Indonesian	**1**
Other	**3**
**Education Level, *n***	
Bachelor’s Degree	**3**
Masters’ Degree	**5**
Medical Practitioner/Clinical Nutritionist	**6**
Doctoral Degree (Ph.D)	**13**
**Years of working experience, *n***	
Less than 5 years	**6**
5 to 10 years	**3**
11 to 20 years	**9**
More than 20 years	**9**

**Table 2:** Summary of Findings.

Socio-Ecological Framework Level	Theme/Sub-Theme	Key Findings
Individual	Awareness about and adherence to a healthy diet	High awareness about, but low adherence to healthy diets in the general population Higher awareness of old guidelines due to simplicity in the messaging Recent guidelines perceived as more complex Overall low adherence to the guidelines
	Impacts of the nutrition transition on dietary preferences	Western /fast-food perceived as unhealthy Marked generational divide in food preferences Older population being generally more health conscious tend to prefer traditional meals Traditional beverages are commonly consumed by all, but new brands of beverages are also becoming popular Nutrition transition makes worse existing nutrition-related health problems
Family/Interpersonal	Changes in family and social eating patterns due to the nutrition transition Home-cooked meals versus meals prepared outside the home	Eating out with family and friends becoming the norm Children’s food preferences prevail over mothers’ choice People acquire new tastes and food preferences over time due to more food options People tend to overeat due to bigger portion sizes People tend to snack more Low level of home-cooking compared to the past due to more women in the workforce, time constraints, complexity of Indonesian dishes, etc
Community/Regional	Cultural meal patterns and their health effects	Religion is a major driver of dietary behaviors Taboo beliefs lead to dietary restrictions of important nutrients for some vulnerable populations Taboo beliefs are firmly entrenched in the food culture
	Impact of the nutrition transition on cultural meal patterns	No difference between traditional and western foods in terms of healthfulness Unhealthy cooking practices contribute largely to high prevalence of CVD risk factors in the population Evolution of traditional staples from root and tubers to rice and noodles Evolution of spices to ultra-process condiments Nutrition transition perceived also as having some benefits: innovative, more options, convenient
Environmental	Impact of the nutrition transition on the food environment Influence of television advertising and social media	Proliferation of foreign food franchises that mimic Indonesian meal patterns Regional differences in food availability being eroded due to increase in the number of online food platforms Restaurants and fast-food shops restrict people’s food choices to unhealthy options Restaurants and food industry use the TV advertising and social media as tools to influence food trends and market unhealthy options Western food franchises are considered safer than local restaurants

## Abbreviations

ASF	Animal Sourced Foods
COREQ	Consolidated Criteria for Reporting Qualitative research
CVD	Cardiovascular Disease
DBM	Double burden of malnutrition
IDGs	Indonesian Dietary Guidelines
IRB	Institutional Review Board
LMICs	Lower-middle income countries
NCDs	Non-communicable diseases
SEM	Socio-ecological model
SES	Socio-economic status
UNDIP	Universitas Diponegoro

## Appendix A. Study Questions Guide on Nutrition Transition

**Icebreaker Question:** If you could eat only one food for the rest of your life, what would it be? Additionally, what is it about this food that you like? (Or, “What is your preferred main-meal time: breakfast, lunch, dinner and why?) **Section 1: Individual-level Factors** 1. First, let us talk about general awareness about diet and health among the Indonesian population 1.1. What are some of their food preferences/food choices that may impact their health, especially increasing risk for obesity, high blood pressure and high cholesterol? 1.2. Where do people typically get advice on diet and health? 1.3. What are the current recommendations for a healthy diet from the Indonesian dietary guidelines? 1.3.1. Are most people aware of the Indonesian dietary guidelines? 1.3.2. Do they typically follow these guidelines? 1.3.3. In your opinion, what gets in the way of adhering to the guidelines? 2. Now, I would like to discuss your views on nutrition transitions in Indonesia. 2.1. Are you familiar with the term “Nutrition Transitions”? 2.2. What are your thoughts concerning nutrition transitions in Indonesia? 2.2.1. Are there any changes in people’s food preference or food choices (including beverages), in recent decades, that may impact people’s health? (If not already addressed above) 2.2.2. What are some food preference/food choices (including beverages) that may increase the risk for obesity, high blood pressure and high cholesterol? **Section 2: Family/Interpersonal Level Factors** 3. Let us talk about how relationships (family, friends) influence the food choice of the population you serve 3.1. In what ways, if any, do family and/or close friends shape the food tastes and preferences of the people you serve? 3.2. Tell me about the usual eating and meal patterns of most families prior to COVID-19 (e.g., communal meals vs. individual meals; one big meal vs. multiple meals; regional and ethnic differences in meal patterns) 4. In what ways have the effect of lifestyle changes in the past 3 or 4 decades impacted family eating patterns 4.1. What about social media and advertisement, etc. (if not already addressed in 1.2.1 above)? 5. Next, let us talk about eating at home versus eating outside 5.1. What are your thoughts on fast food or restaurant food consumption In Indonesia? 5.1.1. What prompt people to eat out (including ordering food online, take out or home delivery), rather than cook/eat at home? 5.2.2. In your opinion, how does this habit influence food choices/preferences and consumption patterns (including snack food, and beverage)? 5.2. What gets in the way of cooking at home? (Ask, only if not already addressed above) **Section 3: Community and Regional Level Factors** 6. Let us talk about eating patterns and health outcomes. 6.1. What, if any, are some common beliefs and/or taboos about foods among the population you serve? 6.1.1. What are your thoughts about why these taboos are there? 6.1.2. In what ways, if any, do these beliefs affect their health outcomes? 6.2. Do you think most people connect their eating patterns to their health outcomes? 6.2.1. If yes, how so? 7. Now, let us talk about how meal preparation practice may impact health, especially CVD risk factors such as obesity, high blood pressure and high cholesterol 7.1. How do cooking practices contribute to CVD risk factors? 7.2. In your opinion, what are some of the most common cooking practices that have the most impactful adverse effect on CVD risk factors? 7.3. What are some of the changes in the composition of traditional diets that may negatively impact people’s health? 7.4. What other thoughts or ideas come to mind concerning eating patterns and CVD risk factors. **Section 4: Global and Environmental Factors** Now we will discuss about the food environment. I will ask about availability, access, and affordability of foods that are generally considered healthy and about the impacts of the nutrition transition on the food environment 8. Let us talk about availability of healthy foods 8.1. Prior to the outbreak of the COVID-19 pandemic, where do people typically buy the food they eat? [Probes: What types of food retail outlets are typically available in most regions of Indonesia: supermarkets, grocery stores, open markets, convenience stores?] 8.2. Comment on the availability of foods that are generally considered healthy, such as fruits, vegetables, whole grains, legumes and nuts in food retail outlets. 8.2.1. Are these foods readily found? 8.3. Is there a variety of such foods available in the retail outlets? 8.3.1. In what significant ways have eating patterns changed in Indonesia over the past three or four decades? 9. Describe the access of these food retail outlets to most consumers, especially in low-income neighborhoods. For instance, prior to the COVID-19 pandemic, are retail outlets that sell healthy food types located near low- income neighborhoods? 10. Comment on the affordability of foods that are generally considered healthy (like fruits, vegetables, whole grains, legumes, nuts and fish), especially for low—income households. Are these food options relatively cheap?
